# The first records of *Spirometra erinaceieuropaei* (Cestoda: Diphyllobothriidae), a causative agent of human sparganosis, in Latvian wildlife

**DOI:** 10.1007/s00436-020-06957-0

**Published:** 2020-11-10

**Authors:** Guna Bagrade, Ivica Králová-Hromadová, Eva Bazsalovicsová, Alžbeta Radačovská, Marta Kołodziej-Sobocińska

**Affiliations:** 1 Latvian State Forest Research Institute “Silava”, Rigas 111, Salaspils, LV-2169 Latvia; 2 Institute of Parasitology, Slovak Academy of Sciences, Hlinkova 3, 040 01 Kosice, Slovakia; 3 Mammal Research Institute, Polish Academy of Sciences, Stoczek 1, 17-230 Białowieża, Poland

**Keywords:** *Lynx lynx*, *Canis lupus*, Parasitic zoonosis, Definitive host, Molecular genotyping, Ribosomal DNA, Mitochondrial DNA

## Abstract

Diphyllobothriid tapeworms of the genus *Spirometra* are causative agents of sparganosis, food-borne zoonotic parasitic disease. They have been recorded in broad spectrum of hosts, including humans, in all continents except Antarctica. *Spirometra* tapeworms have been intensively studied in several Asian countries; however, they have been rather neglected in Europe. The aim of this study was to provide a pilot screening of *Spirometra* spp. in Latvia, where data on sparganosis are not available. Tapeworms morphologically identified as diphyllobothriid species were isolated from grey wolves *Canis lupus* and Eurasian lynxes *Lynx lynx* from Latvia during the hunting periods 2013–2019. The parasites were subjected to molecular genotyping using sequences of the partial large (LSU rDNA; 615 bp) and small (SSU rDNA; 720 bp) subunits of the nuclear ribosomal RNA gene and complete (1566 bp) cytochrome *c* oxidase subunit I gene of the mitochondrial DNA (*cox*1 mtDNA). Analyses of both ribosomal subunits of 13 tapeworms revealed no intraspecific variation within the respective rDNA subunits. On the other hand, sequence analysis of mitochondrial *cox*1 revealed intraspecific polymorphism displayed by 12 *cox*1 haplotypes. Comparison of the current data with sequences of the corresponding DNA regions deposited in the GenBank revealed 99.3–99.5% (LSU rDNA), 99.2% (SSU rDNA) and 99.6–100% (*cox*1 mtDNA) identity of studied tapeworms with *Spirometra erinaceieuropaei,* which provided the first confirmation of this diphyllobothriid tapeworm in Latvia. Since *S. erinaceieuropaei* is probably prevalent in Latvian wildlife and may also occur in other potential host species, further studies are needed in order to acquire complex data on its geographic distribution and transmission in the natural environment of Latvia, as well as on the spectrum of its intermediate, paratenic, and definitive hosts.

## Introduction

Sparganosis is a food-borne zoonosis caused by larval stages (plerocercoids) of diphyllobothriid tapeworms of the genus *Spirometra* (Liu et al. 2015). They have been recorded in humans, domesticated and wild animals in all continents except Antarctica (Scholz et al. 2019). Although several species have been recognized in the genus, the taxonomy of *Spirometra* tapeworms is not fully resolved and requires further clarification (Kuchta et al. 2020). Currently, *Spirometra erinaceieuropaei* (Rudolphi 1819) is the species most often identified in natural environments in Europe (Kondzior et al. 2018; Kołodziej-Sobocińska et al. 2019; Scholz et al. 2019; Kuchta et al. 2020); however, recent data also reported the first confirmation of *S. mansoni* in amphibians in Romania (Kuchta et al. 2020).

The life cycle of *S. erinaceieuropaei* involves copepods (*Cyclops* sp.) as the first intermediate hosts, in which the first stage larva (procercoid) develops. Various vertebrate species of amphibians, reptiles, birds, and mammals may serve as the second intermediate (or paratenic) hosts. In these types of hosts, the second stage larva (plerocercoid), named spargana, develops mainly in subcutaneous tissues and/or muscles (Kuchta et al. 2015). In Europe, almost 40 species of vertebrates were reported as intermediate hosts (e.g. pool frog *Pelophylax lessonae*, marsh frog *Pelophylax ridibundus*, grass snake *Natrix natrix*, common viper *Vipera berus*) (Shimalov 2009; Kondzior et al. 2018; Kuchta et al. 2020) and paratenic hosts (e.g. European badger *Meles meles*, European mink *Mustela lutreola*, American mink *Neovison vison*, European polecat *Mustela putorius*) (Shimalov and Shimalov 2002a; Anisimova 2004; Kołodziej-Sobocińska et al. 2014, 2019; Kuchta et al. 2020). Canids and felids, such as grey wolf *Canis lupus*, Eurasian *Lynx lynx lynx*, wild cat *Felis silvestris,* and cat *Felis catus*, are exclusively definitive hosts (Furmaga 1953; Shimalov and Shimalov 2000; Craig and Craig 2005; Kołodziej-Sobocińska et al. 2018; Kuchta et al. 2020), while raccoon dog *Nyctereutes procyonoides* or red fox *Vulpes vulpes* have been recognized as both definitive and paratenic hosts (Furmaga 1953; Shimalov and Shimalov 2002b, 2003; Kuchta et al. 2020).

While most human cases have been reported from Asia, e.g. China, South Korea, and Thailand (Wiwanitkit 2005; Liu et al. 2015; Hong et al. 2016; Wang et al. 2018), some reports have also been published from Europe, e.g. Italy, Poland, France, Czech Republic, and Germany (Pampiglione et al. 2003; Czyżewska et al. 2019). Humans can be infected either by drinking water contaminated with infected copepods, or by ingestion of plerocercoids present in raw or undercooked meat of second intermediate or paratenic hosts. Adult *Spirometra* tapeworms are also able to mature in the human intestine and can cause a rare disease called spirometrosis, which usually does not result in clinical symptoms (Le et al. 2017). However, the most serious and life-threatening form is proliferative sparganosis (Kikuchi and Maruyama 2020). In Asia, the majority of human cases were most probably connected with the consumption of amphibians and reptiles (Wiwanitkit 2005; Liu et al. 2015), which are not considered to be a source of infection in Europe due to different eating habits. However, wild boar *Sus scrofa* meat cannot be ruled out as a source of infection in Europe (Kołodziej-Sobocińska et al. 2016; Czyżewska et al. 2019), since it has been recorded as a paratenic host of *S. erinaceieuropaei* in Serbia, Belarus, Ukraine, and recently in Poland (Rukavina et al. 1957; Shimalov 2009; Nevolko and Litvinenko 2014; Kołodziej-Sobocińska et al. 2016).

Knowledge about the geographic distribution of *S. erinaceieuropaei* in Europe has been scarce. Since 2000, the tapeworm has been recorded in various wildlife species in Belarus (Shimalov and Shimalov 2000, 2002a, b, 2003; Anisimova 2004; Shimalov 2009), Serbia (Nevolko and Litvinenko 2014), Ukraine (Kornyushin et al. 2011), and most recently in Poland, where *S. erinaceieuropaei* was confirmed by molecular analyses for the first time in Europe (Kołodziej-Sobocińska et al. 2014, 2016, 2018, 2019; Kondzior et al. 2018). It is highly probable that *S. erinaceieuropaei* is also present in other European countries but has been overlooked due to a number of factors. Firstly, some of the hosts are protected species, which can be subjected to detailed parasitological examination only under strict conditions and special permissions. Secondly, the specific location of plerocercoid larvae in subcutaneous tissues and/or muscles of intermediate or paratenic hosts means that the presence of this parasite may be overlooked during standard dissection and parasitological examination, if specific methodology focusing on detection of spargana is not utilized. Finally, taxonomic identification of larval *Spirometra* stages, including *S. erinaceieuropaei*, is complicated owing to the absence of reliable species-specific morphological markers (Kuchta et al. 2015, 2020). Although identification of adult tapeworms based on morphological features is possible (Scioscia et al. 2014), DNA-based genotyping is often recommended in order to confirm the results (Badri et al. 2017; Kołodziej-Sobocińska et al. 2018; Kuchta et al. 2020). The aim of the present work was to acquire additional data on the occurrence of *S. erinaceieuropaei* in Europe, with particular focus on Latvia, where a pilot screening of this zoonotic tapeworm in definitive hosts was performed by molecular genotyping.

## Materials and methods

**Fig. 1:**
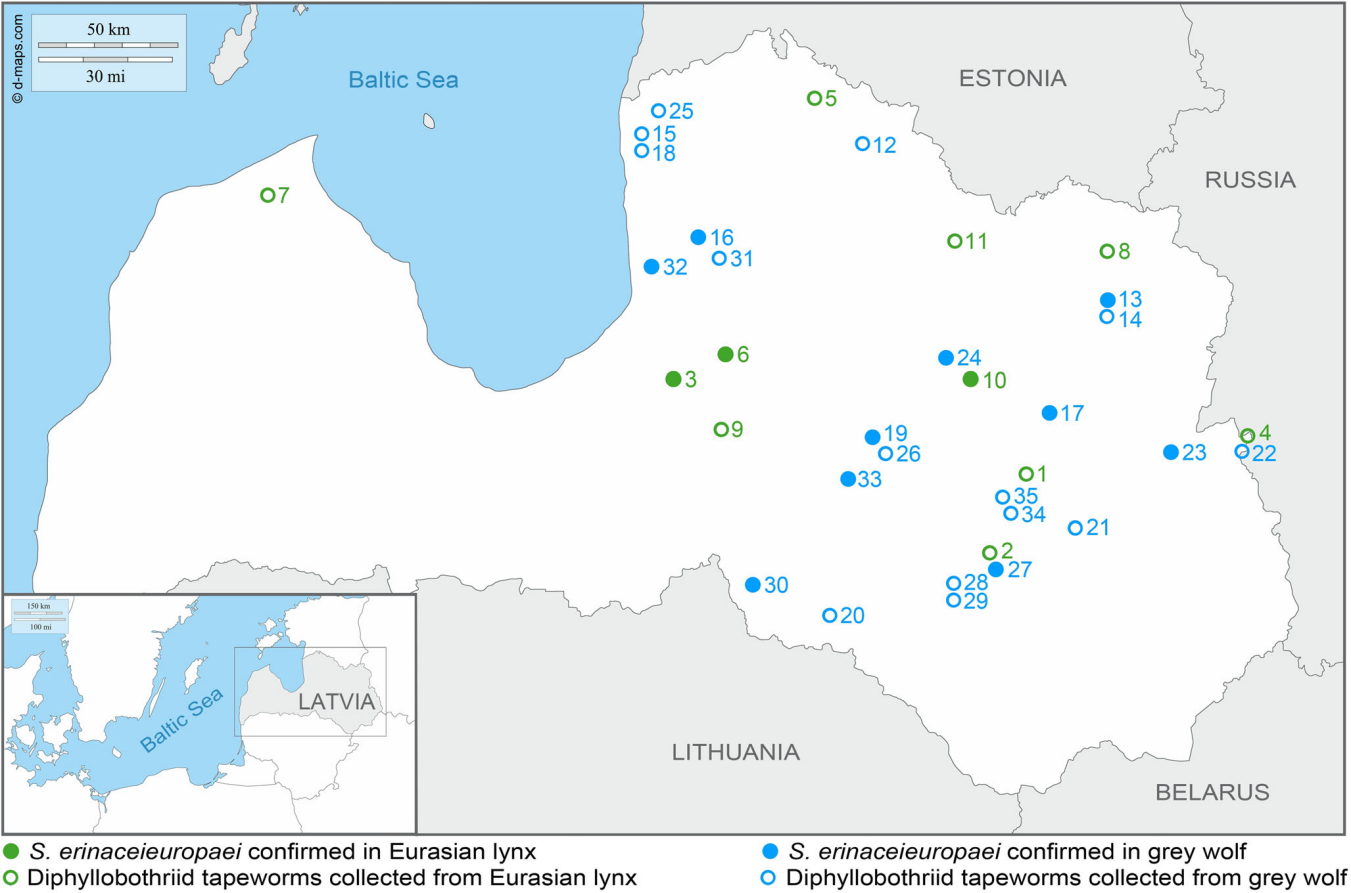
Map of collection sites of diphyllobothriid tapeworms isolated from Eurasian lynxes (green circle) and grey wolves (blue circle) in Latvia with specification of hosts in which *Spirometra erinaceieuropaei* was molecularly confirmed (full circles). Details of individual hosts are provided in Table 1; the numbering of hosts follows the numbers indicated in the column “number of host.” The map was created using Photoshop CS4, version 11.0

The material in the current work originated from the helminthological collection at the Latvian State Forest Research Institute “Silava” in Salaspils, Latvia. In the 2013–2019 hunting seasons, grey wolves (July–March) and Eurasian lynxes (December–March) were hunted within the national legal framework according to the quota allocated by the Latvian Hunting Regulations. A total of 35 hosts, 11 Eurasian lynxes and 24 grey wolves of different ages (juveniles and adults) and sexes (males and females) were examined (Table 1). Figure 1 provides scheme of geographic location of examined Eurasian lynxes and grey wolves in Latvia; all hosts except for one originated from the eastern part of the country.

**Table 1:** Details of hosts and GenBank accession numbers of ribosomal and mitochondrial genes of *Spirometra erinaceieuropaei* from Latvia

No^a^	Code	Age	Gender	Season	Locality	No^c^	LSU rDNA	SSU rDNA	*cox*1 mtDNA	*cox*1 haplotype
1	L1	A	F	2013–2014	Barkava	2	-	-	-	-
2	L2^b^	n.a.	M	2014–2015	Atašiene	1	-	-	-	-
3	L3	n.a.	M	2014–2015	Mālpils	*1*	MT313931	MT313809	MT941770	CO1-Ha2/LV
4	L4	A	F	2015–2016	Goliševa	1	-	-	-	-
5	L5	A	F	2015–2016	Kārķi	1	-	-	-	-
6	L6	A	F	2015–2016	Nītaure	*1*	MT637917	MT637928	MT941769	CO1-Ha1/LV
7	L7	n.a.	M	2016–2017	Dundaga	1	-	-	-	-
8	L8	n.a.	M	2016–2017	Jaunanna	1	-	-	-	-
9	L9	A	F	2016–2017	Madliena	1	-	-	-	-
10	L10	A	M	2017–2018	Cesvaine	*1*	MT637915	MT637921	MT941768	CO1-Ha3/LV
11	L11	J	F	2018–2019	Variņi	2	-	-	-	-
12	W1	n.a.	M	2013–2014	Jērcēnu	2	-	-	-	-
13	W2	J	M	2013–2014	Litene	*1*	MT321262	MT321261	MT951150	CO1-Ha4/LV
14	W3	J	M	2014–2015	Litene	3	-	-	-	-
15	W4	n.a.	F	2014–2015	Salacgrīva	3	-	-	-	-
16	W5	A	M	2014–2015	Umurga	*1*	MT637916	MT637929	MT951146	CO1-Ha5/LV
17	W6	n.a.	M	2014–2015	Lubāna	*1*	MT637923	MT637924	MT951155	CO1-Ha6/LV
18	W7	J	M	2014–2015	Salacgrīva	1	-	-	-	-
19	W8	J	M	2015–2016	Vestiena	*1*	MT637918	MT634698	MT951154	CO1-Ha7/LV
20	W9	J	M	2015–2016	Elkšņi	1	-	-	-	-
21	W10	A	F	2015–2016	Galēni	2	-	-	-	-
22	W11	J	F	2015–2016	Malnava	1	-	-	-	-
23	W12	J	F	2015–2016	Salnava	*1*	MT637926	MT637933	MT951153	CO1-Ha8/LV
24	W13	J	M	2015–2016	Liezēre	*1*	MT637920	MT637934	MT951147	CO1-Ha9/LV
25	W14	n.a.	M	2015–2016	Ainaži	1	-	-	-	-
26	W15	n.a.	F	2015–2016	Vestiena	1	-	-	-	-
27	W16	n.a.	F	2015–2016	Atašiene	*1*	MT637925	MT637935	MT951148	CO1-Ha10/LV
28	W17	A	F	2015–2016	Mežāre	1	-	-	-	-
29	W18	A	F	2015–2016	Mežāre	1	-	-	-	-
30	W19	J	M	2016–2017	Mazzalve	*1*	MT637919	MT637930	MT951152	CO1-Ha1/LV
31	W20	A	F	2016–2017	Umurga	2	-	-	-	-
32	W21	n.a.	F	2017–2018	Skulte	*1*	MT637927	MT637932	MT951149	CO1-Ha11/LV
33	W22	A	F	2017–2018	Klintaine	*1*	MT637922	MT637931	MT951151	CO1-Ha12/LV
34	W23	A	M	2017–2018	Murmastiene	1	-	-	-	-
35	W24	A	F	2017–2018	Murmastiene	1	-	-	-	-

Individuals molecularly confirmed as *Spirometra erinaceieuropaei* are in italics

*L* Eurasian lynx, *W* grey wolf, *A* adult, *J* juvenile, *F* female, *M* male, *n.a.* data not available, *-* data not obtained

^a^Number of host

^b^Roadkill

^c^Number of tapeworms

After complete helminthological dissection, all helminths were taxonomically identified using morphological criteria, preserved in 70% ethanol and deposited in the helminthological collection of the above mentioned institution. For the current work, 44 adult tapeworms were selected from the helminthological collection and subjected to molecular genotyping. The tapeworms were isolated from intestines of Eurasian lynxes and grey wolves immediately after dissection, and they were identified as “diphyllobothriid tapeworms.” Taxonomical identification of diphyllobothriideans is difficult and requires properly fixed and processed material for an accurate morphological description which should be supplemented with molecular genotyping. Since the condition of tapeworms isolated from Eurasian lynxes and grey wolves did not allow reliable morphological description, the material was fixed in ethanol for further identification by molecular genotyping.

Genomic DNA was isolated from 20 mg of tissue of 44 diphyllobothriidean tapeworms using the QIAamp® DNA Kit (QIAGEN, Hilden, Germany) following the manufacturer’s instructions, diluted in deionized water and stored at − 20 °C. Molecular genotyping was performed by Polymerase Chain Reaction (PCR) amplification of a partial fragment of the nuclear large subunit of the ribosomal RNA gene (LSU rDNA) and a partial fragment of the nuclear small subunit of the ribosomal RNA gene (SSU rDNA). LSU rDNA was amplified using the LSU-5 (5′-TAGGTCGACCCGCTGAAYTTAAGCA-3′) and 1500R (5′-GCTATCCTGAGGGAAACTTCG-3′) primers originally described by Olson et al. (2003). Universal primers WormA (5′-GCGAATGGCTCATTAAATCAG-3′) and WormB (5′-CTTGTTACGACTTTTACTTCC-3′) (Littlewood and Olson 2001) were used for amplification of SSU rDNA. In addition, the complete cytochrome *c* oxidase subunit I of the mitochondrial DNA (*cox*1 mtDNA) was amplified using the Diphyllo-Cox1-F (5′-TAGACTAAGTGTTTTCAAAACACTA-3′) and Diphyllo-Cox1-R (5′-ATAGCATGATGCAAAAGG-3′) primers described by Yanagida et al. (2010).

The PCR amplification conditions were 5 min at 94 °C as an initial step, then 30 cycles of 1 min at 94 °C, 1 min at 55 °C, 2 min at 72 °C, and finally 10 min at 72 °C. The PCR products were visualized on a 1.5% agarose gel and purified using exonuclease I and shrimp alkaline phosphatase (Werle et al. 1994). Sequencing was performed by the automatic genetic analyser Applied Biosystems 3130xl (Applied Biosystems, Foster City, California, USA) and the BigDye Terminator v3.1 Cycle sequencing kit (Applied Biosystems). Sequencing of LSU rDNA, SSU rDNA, and *cox*1 mtDNA was performed from both directions using following PCR primers and internal primers. The 5′end of LSU rDNA amplification product was sequenced with LSU-5 primer (primer used for PCR amplification) and two internal primers, 300F (5′-CAAGTACCGTGAGGGAAAGTTG-3′) and 400R (5′-GCAGCTTGACTACACCCG-3′) (Olson et al. 2003). Similarly, the 5′end of SSU rDNA was sequenced with PCR primer WormA and two internal primers 600F (5′-GGTGCCAGCAGCCGCG-3′) and 600R (5′- ACCGCGGCTGCTGGCACC-3′) (Littlewood and Olson 2001). The complete sequence of *cox*1 mtDNA was obtained with two PCR amplification primers Diphyllo-Cox1-F and Diphyllo-Cox1-R (Yanagida et al. 2010) and two internal primers, DCox1-R2 (5′-AAACACCGGCTCACGTAAAG-3′) and Cox1-R3 (5′-CGCAAATGCCGAATAAAGAG-3′) (Kołodziej-Sobocińska et al. 2019). Contiguous sequences were assembled and inspected for errors using the Geneious software (version 10.0.5, Biomatters, Auckland, New Zealand). Obtained sequences were compared with sequences deposited in GenBank using nucleotide BLAST search.

## Results and discussion

Of the 44 diphyllobothriid tapeworms analysed in the current work, PCR products of partial LSU rDNA, partial SSU rDNA and complete *cox*1 were successfully amplified in 13 specimens. PCR amplifications of both ribosomal subunits and mitochondrial *cox*1 were repeatedly carried out under different reaction conditions; however, 31 samples did not yield any amplicons. A possible explanation may be that hunted animals are often provided to the parasitological laboratories at an advanced level of decomposition of the content of their gastro-intestinal tracts. A prolonged period between animal necropsy and parasite fixation may consequently result in a lower level of effectiveness of molecular analyses due to DNA degradation.

Sequence analyses of 615 bp LSU rDNA and 720 bp SSU rDNA of 13 tapeworms revealed identical sequence structure within the respective ribosomal subunit with no intraspecific variation. Comparison of the obtained LSU rDNA sequences with sequences deposited in GenBank revealed 99.5% pairwise sequence identity with LSU rDNA of *S. erinaceieuropaei* (GenBank accession number KY552835) isolated from dog *Canis familiaris* from Australia and 99.3% with *S. erinaceieuropaei* (KY552836) from yellow-spotted keelback snake *Xenochrophis flavipunctatus* from Vietnam (Waeschenbach et al. 2017). Similarly, comparison of the 720 bp fragment of partial SSU rDNA with sequences deposited in GenBank revealed 99.2% identity with respective SSU rDNA region of *S. erinaceieuropaei* (KY552801) isolated from dog from Australia (Waeschenbach et al. 2017).

Sequence analyses of complete mitochondrial *cox*1 sequences (1566 bp) of 13 studied tapeworms revealed intraspecific variation displayed by 1–4 substitutions corresponding to the overall 99.5–99.9% sequence identity. While two individuals (L6 and W19) possessed identical *cox*1 sequence assigned as CO1-Ha1/LV (*cox*1 haplotype no. 1 from Latvia), each of the remaining 11 specimens were characterized by a unique *cox*1 structure corresponding to the specific haplotype CO1-Ha2/LV–CO1-Ha12/LV (Table 1). Comparison of the currently obtained *cox*1 haplotypes from Latvia with complete *cox*1 sequences deposited in the GenBank revealed the highest level of sequence identity (99.7–100%) with *S. erinaceieuropaei* from Poland (MT131826) and Finland (99.6–99.9%; MT131825 (Kuchta et al. 2020).

Sequences of LSU rDNA, SSU rDNA and *cox*1 of all 13 individuals were deposited in GenBank, EMBL and DDBJ databases under accession numbers summarized in Table 1. To conclude, all 13 diphyllobothriidean tapeworms obtained from three Eurasian lynxes and ten grey wolves were molecularly identified as *S. erinaceieuropaei*, providing the first record of this species in Latvia.

Carnivores are important definitive hosts of a wide range of protozoan and metazoan parasites and can play a significant role in the maintenance of different zoonoses (Craig and Craig 2005; Han et al. 2016). Eurasian lynx and grey wolf populations are distributed throughout the entire territory of Latvia. Both populations have increased since the beginning of the 2000s and numbers are estimated within 400–600 for Eurasian lynx and 300–500 for grey wolf (Ozoliņš et al. 2017a, b).

Helminth fauna of Eurasian lynxes from Latvia has been mainly represented by nematodes (*Trichinella britovi*, *Trichinella nativa*, *Toxocara mystax*, *Eucoleus aerophilus* (syn. *Thominx aerophilus*) and *Pearsonema feliscati* (syn. *Capilaria felis-cati*)) and a single tapeworm (*Taenia pisiformis*) and trematode (*Alaria alata*) (Bagrade et al. 2003; Deksne et al. 2016; Ozoliņa et al. 2019). A broader spectrum of helminths has been detected in grey wolves from Latvia. In particular, a single trematode (*Alaria alata*) and several nematodes (*Ancylostoma caninum*, *Crenosoma vulpis*, *Eucoleus aerophilus*, *Pearsonema plica*, *T. britovi*, *Toxocara canis*, and *Uncinaria stenocephala*) were recorded after parasitological examination of grey wolves hunted throughout the country (Bagrade et al. 2009; Deksne et al. 2016). In addition, cyclophyllidean tapeworms represented by *Echinococcus granulosus*, *Echinococcus multilocularis*, *Mesocestoides lineatus*, and several *Taenia* species (*T. crassiceps*, *T. hydatigena*, *T. krabbei*, *T. multiceps*, *T. pisiformis*, and *T. polyacantha*) were found in grey wolves from Latvia. *Dibothriocephalus latus* (syn. *Diphyllobothrium latum*) was detected in a single grey wolf from Latvia as the only representative of diphyllobothriidean tapeworms (Bagrade et al. 2009). However, the taxonomic identification of all mentioned helminths reported from grey wolves and Eurasian lynxes from Latvia was based only on their morphology and no molecular genotyping was performed.

Since taxonomic identification of diphyllobothriidean tapeworms based only on morphology is difficult (Kuchta et al. 2015, 2020) and the previously published occurrence of *D. latus* in a grey wolf in Latvia (Bagrade et al. 2009) was not confirmed by molecular methods, further studies focused on detailed morphological descriptions supplemented with molecular genotyping of diphyllobothriids are highly recommended for their more accurate taxonomic identification in Latvian wildlife. *Dibothriocephalus latus* is evidently circulating in the natural environment in Latvia because its larval stages, plerocercoids, were detected during ichthyoparasitological examinations in several fish species (Kirjušina and Vismanis 2007). In addition, human cases of diphyllobothriosis (up to 10 cases over 2010–2014) have been recorded in patients from Latvia by the Centre for Disease Prevention and Control (https://spkc.gov.lv). However, it is evident that more data on the -occurrence, prevalence, geographic distribution, and a spectrum of intermediate and definitive hosts of *D. latus* in Latvia are required for the up-to-date knowledge on circulation of this most frequent causative agent of diphyllobothriosis in Europe.

It is not possible to conclude if our findings represent a recent introduction of *S. erinaceieuropaei* to Latvia, or if this tapeworm has been present in the Latvian environment for a prolonged period. Since sparganosis has been confirmed in neighbouring Belarus (Shimalov and Shimalov 2000, 2002a, b, 2003; Shimalov 2009) and geographically proximal Poland (Kołodziej-Sobocińska et al. 2014, 2016, 2018, 2019) and Finland (Lavikainen et al. 2013; Kuchta et al. 2020), it seems highly probable that *S. erinaceieuropaei* had been present in Latvia for a longer time.

The current study provided initial data on spirometrosis in Latvia, but reveals several perspectives for future studies. Investigating the role of particular intermediate and paratenic hosts of *S. erinaceieuropaei* is highly important for understanding its life cycle and dispersal in the natural environment in Latvia. In addition, the potential zoonotic risk of human sparganosis connected with wild boar meat consumption or drinking contaminated water should be examined from a medical health perspective.
